# Calculation of continuous reference intervals for biological parameters exhibiting strong age‐dependent level changes: Its application to glycosaminoglycans and sialic acid in urine

**DOI:** 10.1002/jmd2.12448

**Published:** 2024-10-01

**Authors:** Carlos Emilio Rodríguez, Mette Diswall, Anders Olsson, Torleif Jonsson, Eva Johansson, Maria Blomqvist

**Affiliations:** ^1^ Department of Clinical Chemistry Sahlgrenska University Hospital Gothenburg Sweden; ^2^ Department of Laboratory Medicine, Institute of Biomedicine University of Gothenburg Gothenburg Sweden

**Keywords:** age‐dependent reference intervals, continuous reference intervals, glycoproteinosis, glycosaminoglycans, mucopolysaccharidosis, sialic acid

## Abstract

Glycosaminoglycan (GAG) and sialic acid (total and free) assays are used as first‐line screening tests for the diagnosis of mucopolysaccharidoses and glycoproteinoses, respectively. There is a pronounced age‐dependent variation in the urinary concentrations of these metabolites in the normal population, and the stratification of the reference values into discrete age ranges may lead to an undesirably high number of false‐positive or false‐negative results. The aim of this study was to design a method for calculating continuous reference intervals as a function of age and its application to the analysis of GAGs and sialic acid (total, free, and conjugated) in urine. In the postpubertal period, concentrations of urinary GAGs and sialic acid have reached a plateau, so a traditional calculation of the reference range in this specific age group was considered appropriate. In the prepubertal period, a nonlinear regression performed with the Excel add‐in Solver was used to fit the logarithmized concentrations of the controls to a curve that represents the mean values as a function of age. A uniform distribution of the residuals was obtained, which allowed the calculation of the reference intervals by adding the values of their 2.5 and 97.5 percentiles to the independent variable of the regression curve to calculate the upper and lower reference curves. The main advantages of the developed method are (1) a reduction in the number of control samples needed to obtain adequate reference intervals and (2) an improvement in the reliability of diagnostic screening by reducing the uncertainty generated by the gaps in the traditional age‐stratified method.

**Synopsis**

Continuous reference intervals for glycosaminoglycans and sialic acid in urine.

## 1 INTRODUCTION

Mucopolysaccharidoses (MPS) and glycoproteinoses are two groups of inherited metabolic diseases caused by the absence or malfunctioning of lysosomal enzymes that are involved in the catabolism of glycosaminoglycans (GAGs) and glycoproteins, respectively.^1^ Analysis of GAGs and sialic acid in urine is employed for their diagnosis. For both parameters, there is a pronounced variation in the normal concentrations in urine as a function of age from the newborn period to puberty and adulthood.

The calculation of normal reference intervals for biological parameters that vary with age has traditionally been resolved through stratification in discrete age intervals.^2^, ^3^, ^4^, ^5^ The reference intervals are calculated for each age range using the approximation that no dependence on age exists within the range. The quantity and dimensions of the age intervals are conditioned by the minimum number of controls that each interval must include. International guidelines recommend at least 120 samples per interval if nonparametric methods must be applied due to non‐Gaussian distributions.^6^, ^7^ However, it can be difficult to obtain this number of controls for every interval, and it is common to use fewer controls as well as intervals wider than desirable. Thus, this approach generates reference values with a significant degree of uncertainty, and reanalysis or additional tests are commonly needed to confirm or rule out diagnosis.

A continuous reference interval is the ideal solution to these problems. Some groups have used continuous age‐dependent reference ranges by means of percentile charts^8^, ^9^ or applied a parametric regression model where the covariate age goes into the regression model as a linear function of the square root of age,^10^ but both methods required a large cohort of samples.

The objective of this work was to obtain continuous reference intervals for the analysis of GAGs and sialic acid in urine with a method that can be carried out with a more limited number of controls. The main difficulties that we had to overcome were choosing a function capable of fitting the data correctly and finding a suitable nonlinear regression algorithm that achieves convergence for the variables of the function, thus generating residuals with a uniform distribution throughout the whole age range.

## 2 MATERIALS AND METHODS

Urine control samples were obtained from employees and patients at the Clinical Chemistry Department, Sahlgrenska University Hospital, Gothenburg, Sweden. Patients with MPS or glycoproteinoses were excluded. The calculations of the new reference intervals were performed after a method change for both analyses, GAG and sialic acid. Some biobanked samples were reanalyzed but the main part of the control material was new samples, and the recruitment period elapsed approximately 1 year. Urine samples were kept at −20°C and deidentified when included in the study. All the urines are centrifuged, and the supernatant is separated from the sediment. To correct for the effect of dilution by hydration the concentrations of GAG and sialic acid are expressed per mole of creatinine. Highly dilute urine samples with creatinine concentrations below 1 mmol/L were discarded because they may lead to falsely elevated results.^11^ On the contrary, we are not aware that highly concentrated urine can lead to falsely reduced or elevated values, and we have not observed this problem in our daily practice with these biological parameters.

GAG analyses in urine were performed with the DMB (dimethylmethylene blue) method as previously described by Andrade et al.^12^ The internal quality controls (Control Special Assays in Urine SAU‐01.1 and SAU‐01.2, MCA Laboratories, Winterswijk, Netherlands) showed good precision with CVs <4% for the low QC level and <3% for the high QC.

Quantitation of free and total sialic acid (FSA and TSA) in urine was performed by LC–MS/MS (the method is described in detail in Data S6). The internal quality controls (homemade, low QC consists of pooled urine of control samples and high QC consists of pooled urine of patient samples) showed good precisions with CVs <4.5% for the two QC levels of both parameters (TSA and FSA).

Our laboratory is participating in the following ERNDIM (Quality Assurance in Laboratory Testing for IEM) control programs: MPS Scheme, Quantitative Special Assays in Urine and Diagnostic Proficiency Scheme in Urine.

The creatinine in urine was analyzed by the Abbott enzymatic creatinine method on an Alinity c instrument (Abbott, Chicago, IL, USA).

SPSS version 28.0.1.1 (IBM SPSS Statistics, Chicago, IL, USA) was employed to perform the statistical analyses.

### 2.1 GAG samples

A set of 186 controls between the ages of 0 and 63 years was subdivided into the prepubertal period (<14‐year old, *n* = 146) and the postpubertal period (>14‐year old, *n* = 40). The method described here was then validated for GAG analysis. For this purpose, 42 additional controls between the ages of 0 and 14 years were used to validate the derived reference intervals.

### 2.2 TSA and FSA samples

A set of 140 controls between the ages of 0 and 61 years was subdivided into the prepubertal period (<14‐year old, *n* = 88) and the postpubertal period (>14‐year old, *n* = 52).

### 2.3 Calculation of reference intervals in the age range of >14 years

In the postpubertal period, urinary concentrations of GAGs and sialic acid have reached a plateau, making it possible to carry out a standard nonparametric calculation of the reference range in this specific age group. The Kolmogorov–Smirnov test with the Lilliefors significance correction and the Q‐Q plot were calculated to evaluate the normality of the data distributions. The null hypothesis was clearly rejected for FSA (*p* = 0.009) and was close to significance at 95% for GAG (*p* = 0.053) and TSA (*p* = 0.086). Conjugated sialic acid (CSA) was calculated by subtracting FSA from TSA, and this parameter showed a more Gaussian distribution (*p* = 0.200). The GAGs, TSA, and FSA concentrations were logarithmized to transform the data into Gaussian distributions with *p* values clearly far from significance (*p* = 0.200). All reference intervals were then calculated with parametric method (see Data S1). Confidence intervals (90% CI) of the reference intervals limits were calculated according to IFCC recommendations.^7^

### 2.4 Calculations of continuous reference intervals in the age range of <14 years

The calculations and plots for all the parameters are in Data [Link], [Link], [Link], [Link].

The detailed description of the method employed to calculate the continuous reference intervals in the prepubertal period is as follows.

The ages of the individuals were expressed in fractions of years with the Excel function YEARFRAC from the dates of birth and sampling.

A nonlinear regression of the logarithmized values to Equation (1) was performed:

(1) $$y=a\times \ln\left(x+d\right)+b,$$

where *a*, *b*, and *d* are variables to be calculated by the regression, *y* is the natural logarithm of the concentrations per mole of creatinine, and *x* is age. The term *d* determines the value of the vertical asymptote of the curve. This term was introduced to allow negative values of the asymptote, and by means of this procedure, the curve crosses the ordinate axis at a finite value. This improves the fit of the regression by avoiding the singularity at a concentration close to zero. With this procedure, the skew is eliminated, and the distribution of the residuals is uniform across all ages, even at very low ones.

The main complication with the addition of the *d* term is that nonlinear regression to this function is not supported by the standard Excel software. The Excel add‐in Solver was used to perform a nonlinear regression by an iterative least‐squares fitting routine as described by Brown.^13^

The variables calculated by the regression determine the fitted curve from which the residuals are calculated.

The Kolmogorov–Smirnov one‐sample test with the Lilliefors correction, the Shapiro–Wilks test, the Q‐Q plot, and the histogram of the values were used to test the normality of the distributions of the residuals.

The 2.5 and 97.5 percentiles of the residuals' distribution were calculated, and their values were added to the independent term *b* of the fitted curve to obtain the upper and lower reference interval curves.

Finally, the antilogarithms of the three equations were calculated to express the fitted curve and the upper and lower reference interval curves in terms of g GAGs/mol creatinine or mmol SIA/mol creatinine versus age.

### 2.5 Nonlinear regression with the Excel add‐in Solver

The nonlinear regression follows in detail the method described by Brown.^13^ The following points are addressed for clarification of the nonlinear regression with the Excel add‐in Solver:

- To activate the Solver add‐in in Excel 2010 and later versions, follow the sequence File > Options > Add‐Ins. In the Manage box, select Excel Add‐Ins and click “Go.” Then, in the Add‐Ins available box, select Solver Add‐In. If it is not available, click “Browse” to locate it. If the Solver Add‐In is not installed on your computer, install it. When the Solver Add‐In is activated, the command is available in the tab Data > Analysis.
- The Excel files in the Supporting information are configured according to the European format with a comma as the decimal separator.
- In Excel, it is not possible to use the letters “r” or “c” to name a cell because it comes into conflict with Excel's RC nomenclature. That was the reason for choosing the variables *a*, *b*, and *d* in Equation (1).
- In the option “Set Objective” of the “Solver Parameters” window, the cell with the numerical value of R2 was selected to maximize the coefficient of determination.
- The cells with the numerical values of the variables *a*, *b*, and *d* were chosen in the box “By Changing Variable Cells.”
- The value of the variable *a* was constrained between 0 and −1 in the window “Solver Parameters”: *a* ≤ 0 and *a* ≥ −1.
- The box “Make Unconstrained Variables Non‐Negative” was activated.
- “Non‐linear GRG” (generalized reduced gradient) was selected as “Solving Method,” and the “Options” window was configurated as follows:
- Tab “All methods”: Precision: 0,000001, “Use automatic scaling”: checked, 100 iterations, and a max time of 100 s.
- Tab “Non‐linear GRG”: Convergence 0,00001, “Derivator”: Next. “Use Multistart”: checked, population size for the GAG parameter: 146, population size for the sialic acid parameters: 88, and a “Random Seed” of 10. “Require Bounds of Variables”: unchecked.
- The running of the Solver add‐in returns the results of the variables *a*, *b*, and *d*, as well as the other parameters described by Brown^13^ (Mean_of_*y*, *df*, *SE* of *Y*, R2, Critical *t*, and CI).

### 2.6 Validation of continuous reference intervals

The method was then validated for the GAG parameter using 42 additional controls from individuals aged <14 years. The values were logarithmized, and the residuals were calculated from the regression curve obtained with the 146 original controls. Their distribution was compared with that of the original controls using the two‐sample Kolmogorov–Smirnov test. In this test, the statistic is $D_{n,m}=\underset{x}{\sup}\left|F_{1,n}\left(x\right)-F_{2,m}\left(x\right)\right|$, where *F*_1,*n*_ and *F*_2,*m*_ are the empirical distribution functions of the controls and validation controls, respectively, and sup is the supremum function. The null hypothesis is rejected if the statistic $D_{n,m}>C\left(\alpha \right)\;\sqrt{\frac{n+m}{n\times m}}$, where $C\left(\alpha \right)=\sqrt{-\ln\left(\frac{\alpha }{2}\right)∙\frac{1}{2}\;}=1.358$ for *α* = 0.05, *n* = 146 (number of primary controls), and *m* = 42 (number of validation controls). The null hypothesis is that the distributions of both populations do not differ.

## 3 RESULTS

### 3.1 Reference intervals in the age range of >14 years

The medians and the reference intervals (in brackets: 90% confidence interval of the limits) obtained in the age range of >14 years for all the studied parameters are as follows: GAG: 2.6: 1.3 (1.1–1.5)–5.1 (4.4–5.9) g/mol creatinine; TSA: 22.5: 12.6 (11.2–14.1)–40.3 (36.0–45.2) mmol/mol creatinine; FSA: 5.7: 3.2 (2.9–3.6)–10.0 (9.0–11.2) mmol/mol creatinine; and CSA: 17.5: 6.8 (4.7–8.9)–28.2 (26.1–30.3) mmol/mol creatinine.

The calculation of the reference intervals and the respective confidence intervals can be found in the Data S1. The file has a tab for each parameter, and the results of the statistical study carried out with the SPSS program are inserted as figures.

### 3.2 Continuous reference intervals in the age range of <14 years

**FIGURE 1:**
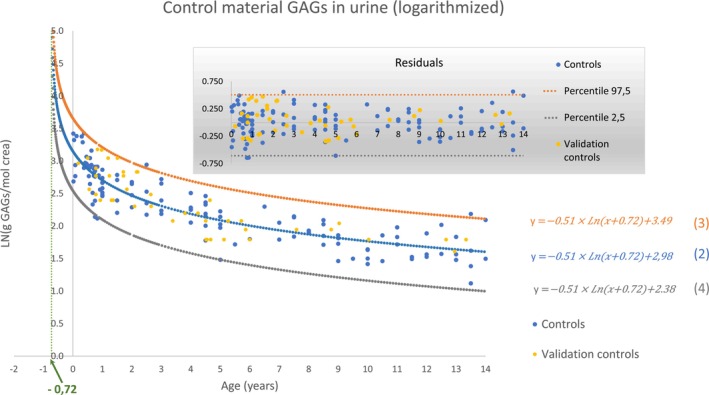
Result of the nonlinear regression for the age range of <14 years. In the graph with the shaded area, the values of the residuals are plotted. The validation controls were not included in the nonlinear regression. They were used only to validate the method.

**FIGURE 2:**
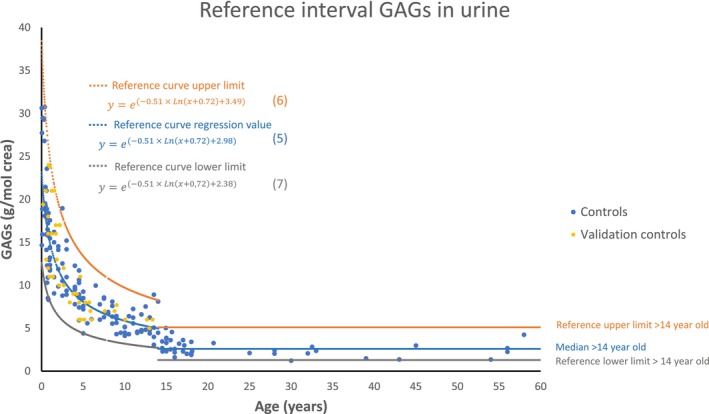
Age‐dependent reference interval for the GAG parameter in urine. GAG, glycosaminoglycan.

The use of a nonlinear regression to the logarithmic function (Equation (1)), with the data derived from the logarithmic transformation of the GAGs and SIA concentrations, results in a uniform distribution of the residuals all along the abscissa axis (see the graph “residuals” in Figure 1 for the GAG parameter). This allows the calculation of the reference interval based on the distribution of the residuals. The curves that correspond to the upper and lower limits (given by equations (3) and (4) in Figure 1) were obtained by the addition of the 2.5 and 97.5 percentiles of this distribution to the independent term *b* of equation (2) in Figure 1. Finally, the natural antilogarithm of equations (2), (3), and (4) was performed to obtain the reference interval curves in g GAGs/mol creatinine (equations (5), (6), and (7) in Figure 2) and in mmol/mol creatinine for TSA, FSA, and CSA (see Tab “Antilog Ref.int alla” of Data [Link], [Link], [Link], [Link]).

Figure 2 shows the reference interval for the GAG parameter over the entire age range. The gap of the reference intervals at 14 years reflects the abrupt arrest of growth around this age, which is noticeable despite the interindividual variability.

### 3.3 Validation of the continuous reference intervals for GAG

The goodness‐of‐fit comparison of the two‐sample Kolmogorov–Smirnov test resulted in an asymptotic significance of 0.925 and a value of $D_{n,m}=0.096$ (see Table 1). This value is lower than the parameter $C\left(\alpha \right)\;\sqrt{\frac{n+m}{n\times m}}=0.175$, and the null hypothesis is therefore not rejected. These results point to a distribution of the residuals of the validation controls (represented in yellow in Figure 1) that does not differ from the distribution of the residuals of the original controls (in blue).

**TABLE 1:** Two‐sample Kolmogorov–Smirnov test for the comparison of the residuals of the original controls (*n* = 146) with those of the validation controls (*n* = 42) for the GAG parameter.

Most extreme differences (*D*_*n*,*m*_)	
Absolute	0.096
Positive	0.096
Negative	−0.060
Kolmogorov–Smirnov *Z*	0.0548
Asymp. sig. (two‐tailed)	0.925

Abbreviation: GAG, glycosaminoglycan.

## 4 DISCUSSION

The high concentration of metabolites present in the urine at low ages is most likely a consequence (at least in part) of the high rate of renal growth at this stage of life. It makes sense that metabolites may leak into the urine when the size and morphology of the kidney are changing rapidly. The increase in glomerular filtration rate and tubular excretory capacity in humans through childhood is a well‐known fact,^14^, ^15^ but there are insufficient data on the growth rate of the kidney as a function of age in humans. There are detailed data on the growth of the renal cortex and the kidney in rats,^16^ showing a marked growth rate until puberty, and from this stage, these organs remain practically unchanged for the rest of their lifespan.

Although renal growth may not be strictly identical to growth in height, this comparison can serve as an additional guide to choosing the division point between the prepubertal curved reference interval and the postpubertal fixed reference interval. Data on the growth rate in height in humans are available,^17^ and this growth rate (expressed in mm/month) declines almost exponentially in the first years of life and then rises slightly again during puberty, reaching a top between 11 and 12 years of age for women and between 13 and 14 years of age for men. This behavior agrees with that of the excretion of the metabolites studied in the present study. The choice of 14 years of age implies a discontinuity (gap) in the reference interval at this age, which applies to all the parameters studied here, and the magnitude of the discontinuity is similar to that obtained by the traditional method with age stratification.

Logarithmic transformation is a widespread practice in the calculation of reference values of populations with a skewed distribution.^18^, ^19^, ^20^ The goal of this transformation is to reduce the skewness or even transform the data from a non‐Gaussian to a Gaussian distribution. When a parameter varies with age, it is not possible to know the skewness of the distribution, which depends mostly on the age distribution of the controls included in the dataset. That is why we, by default, chose to perform a logarithmic transformation of the individual values.

For a nonlinear regression, it is crucial to choose an equation with the ability to fit the data correctly. Of the standard trendlines of the Excel program, the logarithmic function best fitted the data. However, in its basic form, this function has a vertical asymptote at the origin of the abscissa axis, causing a skewed distribution of the residuals when age approaches zero. For this reason, a variable was added (variable *d* in Equation (1)) that determines the position of the asymptote. This variable was constrained to negative values in the regression, not only because renal growth already exists during the fetal phase but also because metabolites must have a finite value. With this approach the curves cross the ordinate axis at a finite value, the singularity at ages close to the origin of the abscissa is avoided, and the distribution of the residuals becomes uniform even at very low ages.

Initially, the SPSS algorithms Levenverg–Marquardt and sequential quadratic programming were used to perform the nonlinear regressions, but they failed to achieve convergence or give a good fit of the results. These algorithms work better when all the data fit a curve with low dispersion of the residuals. In the case at hand, the natural interindividual biological variation of the data produces a higher dispersion of the residuals. Under these conditions, the GRG algorithm used by the Excel Solver add‐in is much more robust in finding convergence and obtaining the optimal solution that minimizes the sum of squares of the residuals. This algorithm has a series of effective program subroutines that find the conditions for continuing the iterations when the Newton iterative algorithm does not find convergence,^21^, ^22^ and since the beginning of its development, it has been used successfully to solve optimization problems in different fields.^23^, ^24^, ^25^

The reduction in the number of normal controls needed to calculate adequate reference intervals is one of the main advantages of the method described here. If a nonparametric method is used, international guidelines recommend at least 120 controls per age interval for calculating a reference interval.^6^, ^7^ If a population can be considered to have a Gaussian distribution or if the data can be transformed to form a Gaussian distribution, a smaller number of controls are needed. However, in clinical practice, in cases in which the availability of controls is limited (which is almost always the case for pediatric samples), it is common to reduce this number to between 30 and 40 controls in a dataset, assuming a Gaussian distribution. However, in highly stratified reference intervals, it can be difficult to have even 20 controls for the youngest ages, and unfortunately, at a young age more stratification is desirable. The biological parameters included in this study require stratification into five to seven age groups^2^, ^3^, ^4^, ^5^, ^26^, ^27^, ^28^ at <14 years of age. With the new method presented here, adequate reference intervals can be achieved with a single interval in which 120 (optimum) or at least 40 (viable) controls are sufficient, with the only requirement of a uniform age distribution throughout the range.

The analysis of GAGs and sialic acid in urine is commonly used as a first‐tier screening approach when a mucopolysaccharidosis or glycoproteinosis disorder is suspected. A good specificity and sensitivity of those assays is very important, since confirmation of diagnoses requires to perform new biochemical and genetic analyses, which are expensive and time‐consuming. In Figure S7 we have presented the GAGs concentration of MPS patients when they were diagnosed in our laboratory along with controls and reference intervals. Although most patients have a very high increase in concentrations, some of them are just above the upper limit of the reference range, especially patients with Morquio A (MPS IVa).

In Figure S8 the areas of discrepancy between the discrete intervals and the continuous interval can be seen for the GAG parameter in the age interval <14 years. The elimination of the areas of bias in the discrete intervals caused by the dependence of the parameter with age improves the diagnostic reliability for these disease groups and thereby reduce the time to diagnosis by avoiding unnecessary laborious procedures.

### 4.1 Concluding remarks

The method for calculating continuous reference intervals described in this study eliminates the problems arising from the discontinuous stratification of reference ranges. In addition to the need for fewer controls for the calculations, the diagnostic specificity and sensitivity will be improved, thereby reducing diagnostic failures or delays.

## AUTHOR CONTRIBUTIONS

Carlos Emilio Rodríguez conceived the presented idea, developed the theory, performed the calculations, and wrote the manuscript. Mette Diswall assisted with the development and description of analysis method, measurements, and revised all the graphic material. Anders Olsson provided critical feedback and helped supervise the project and manuscript. Torleif Jonsson assisted with the development and description of analysis method, measurements, and collection of data. Eva Johansson assisted with the development and description of analysis method, measurements, and collection of data. Maria Blomqvist provided critical feedback and helped supervise the project, analysis, and manuscript.

## CONFLICT OF INTEREST STATEMENT

The authors declare no conflicts of interest.

## ETHICS STATEMENT

This study was carried out in accordance with the Swedish Ethical Review Authority (Etikprövningsmyndigheten) and the International Code of Medical Ethics of the World Medical Association (Declaration of Helsinki).

## Supporting information


**Data S1.** Reference intervals for GAGs, TSA, FSA, and CSA over 14‐year old.

**Data S2.** Calculation of the reference interval for GAGs under 14‐year old.

**Data S3.** Calculation of the reference interval for TSA under 14‐year old.

**Data S4.** Calculation of the reference interval for FSA under 14‐year old.

**Data S5.** Calculation of the reference interval for CSA under 14‐year old.

**Data S6.** Method for the analysis of sialic acid in urine.

**Figure S7.** Reference interval of GAGs in urine including values from controls and MPS patients. MPS IH (Hurler syndrome), MPS II (Hunter syndrome), MPS VI (Maroteaux‐Lamy syndrome), MPS IIIa (Sanfilippo A syndrome), MPS IIIb (Sanfilippo B syndrome), MPS IIIc (Sanfilippo C syndrome), and MPS IVa (Morquio A syndrome).

**Figure S8.** Discrete and continuous reference intervals of GAGs. The striped areas are the areas of discrepancy between the discrete intervals and the continuous interval.

## Data Availability

Data supporting the results reported in the article can be found in the Supporting information.
