# Supplementary material for: Calculation of continuous reference intervals for biological parameters exhibiting strong age‐dependent level changes: Its application to glycosaminoglycans and sialic acid in urine
Source: JIMD Rep. 2024 Oct 1;65(6):442–9. doi: 10.1002/jmd2.12448 (PMC11540561; doi:10.1002/jmd2.12448)
Supplement: Supplementary file 6 — Data S6. Method for the analysis of sialic acid in urine. [file JMD2-65-442-s005.docx]

**Supplementary Material Table 1.**

MS/MS parameters (compound and internal standard)

| ***UPLC parameters*** | | |  | ***Mass spectrometry parameters*** | | | | |  |
| --- | --- | --- | --- | --- | --- | --- | --- | --- | --- |
|  | |  | Mass spectrometer | |  | Waters Xevo TQS | | |  |
| Column | | Acquity UPLC BEH C18 | Ionization mode | |  | Negative | | |  |
| Column T | | 40 °C | Capillary voltage | |  | -2.0 kV | | |  |
| Weak wash | | 0.1% formic acid in water | Source T | |  | 150 °C | | |  |
| Strong wash | | Acetonitrile:water:formic acid (90:9.5:0.5, v:v:v) | Desolvation T | |  | 500 °C | | |  |
| Mobile phase A | | 5 mM ammonium formate, 0.2% formic acid in water | Cone gas flow | |  | 150 °C | | |  |
| Mobile phase B | | Acetonitrile | Dwell time | |  |  | | |  |
| Gradient | | A flow rate of 0.3 mL/min was used during the whole run time. An initial rate of 5% B. From 0.5 min to 1.0 min, a linear gradient to 30% B. From 1.0 min to 1.3 min, a linear gradient to 99% B. The system returns to 5% B from 2.2 min to 2.4 min and then holds an isocratic flow of 5% B to 3.0 min. | Desolvation gas flow | |  | 1000 L/h | | |  |
| Injection volume | | 2 μl |  | |  |  | | |  |
| Injection mode | | Partial loop with needle overfill |  | |  |  |  |  | |
| Autosample temperature | | 10°C |  | |  |  |  |  | |
| Total run time | | 3.5 min (injection to injection) |  | |  |  |  |  | |
|  | |  |  | |  |  |  |  | |
|  | |  |  | |  |  |  |  | |
|  | ***Compound information*** | | | | | | | | |
| Compound | | Parent ion (m/z) | Daughter ion  (m/z) | | Dwell (s) | Cone voltage | Collision energy | Retention time (min) | |
| Sialic acid | | 308.11 | 87.0 | | 0.050 | 15 | 15 | 0.88 | |
| ^13^C_3_-Sialic acid | | 311.12 | 90.0 | | 0.050 | 15 | 15 | 0.88 | |

Sample preparation

Urine (25 µl) was pipetted into Eppendorf tubes, and an internal standard (25 µl) was added to each tube.

Free sialic acid: 100 µl ultrafiltered water was added followed by 850 µl acetonitrile. Tubes were mixed and centrifuged (17000 g, 6 min).

Total sialic acid: 100 µl 62.5 mmol/L sulfuric acid (aq) was added, and tubes were mixed and subjected to hydrolysis (80 °C, 60 min). Further, 850 µl acetonitrile was added, and tubes were mixed and centrifuged (17000 g, 6 min).

Following centrifugation, 50 µl of the supernatants (containing free and total sialic acid) was transferred to a sample plate and diluted with 450 µl ultrafiltered water. The plate was shaken, centrifuged (3000 g, 5 min), and subjected to LC-MS/MS-analysis.

Quality control samples

Urine samples of appropriate levels were pooled to approximately 250 µmol/L (QC low level) and 700 µmol/L (QC high level).

Calibration curve and internal standard:

A six-level calibration curve was prepared by dissolving *N*-acetylneuraminic acid (product number 19023, purity 100%, Sigma-Aldrich) in ultrafiltered water to 10, 20, 50, 150, 400, and 1000 µmol/L. Likewise, an internal standard was prepared by dissolving ^13^C_3_-*N*-acetylneuraminic acid (product number 649694, purity ≥99%, Sigma-Aldrich) in ultrafiltered water to a concentration of 750 µmol/L.

LC-MS/MS analysis:

A Waters Xevo TQS mass spectrometer equipped with an Acquity UPLC separation system (Waters, Wilford, MA, USA) was used. Mass spectrometric and chromatographic conditions are specified in Supplementary Material Table 1. Evaluation of the data and quantification were conducted using MassLynxTM software version 4.2 (Waters, Wilford, MA, USA).

Method validation

Precision was determined in QC samples (five replicates measured at five separate working days, n = 25). The intra-/inter-series CV (%) at low QC level was 2.7/3.2% (free sialic acid) and 2.3/2.5% (total sialic acid). At high QC level, the intra-/inter-series CV (%) was 3.8/4.2% (free sialic acid) and 2.0/2.2% (total sialic acid).

The lower limit of the quantification of the method was measured at the lowest level of the calibration curve (five replicates measured at three separate working days, n = 15), yielding 9.71 ± 0.743 µmol/L with a CV of 7.7% (free sialic acid) and 9.8 ± 0.7 µmol/L with a CV of 10.0% (total sialic acid). For all analyses, the signal-to-noise ratio of the analyte peak was ≥10.

The linearity was evaluated across the calibration range with mean correlation coefficients, *R^2^*, >0.99 (n = 5). The percent deviation (the difference between the observed and the predicted concentrations) was <4% for all calibrator levels (n = 5).

After the addition of analyte (80 µmol/L) to ten individual donor urine samples, the average recoveries were 102% (free sialic acid) and 100% (total sialic acid). The accuracy of total sialic acid evaluated in EQA samples (ERNDIM Special Assays in urine) in duplicates at three concentration levels (125, 200, and 275 µmol/L) was on average 99%. Both recovery and accuracy were determined as measured level/(spiked amount + baseline level).

Conjugated sialic acid was calculated by subtraction of free sialic acid from total sialic acid.

The creatinine in urine was analyzed by the Abbott enzymatic creatinine method on an Alinity c instrument (Abbott, Chicago, IL, USA).

SPSS version 28.0.1.1 (IBM SPSS Statistics, Chicago, IL, USA) was employed to perform the statistical analyses.
