# Supplementary material for: Calculation of continuous reference intervals for biological parameters exhibiting strong age‐dependent level changes: Its application to glycosaminoglycans and sialic acid in urine
Source: JIMD Rep. 2024 Oct 1;65(6):442–9. doi: 10.1002/jmd2.12448 (PMC11540561; doi:10.1002/jmd2.12448)
Supplement: Supplementary file 7 — Figure S7. Reference interval of GAGs in urine including values from controls and MPS patients. MPS IH (Hurler syndrome), MPS II (Hunter syndrome), MPS VI (Maroteaux‐Lamy syndrome), MPS IIIa (Sanfilippo A syndrome), MPS IIIb (Sanfilippo B syndrome), MPS IIIc (Sanfilippo C syndrome), and MPS IVa (Morquio A syndrome). [file JMD2-65-442-s008.pptx]

## Slide 1
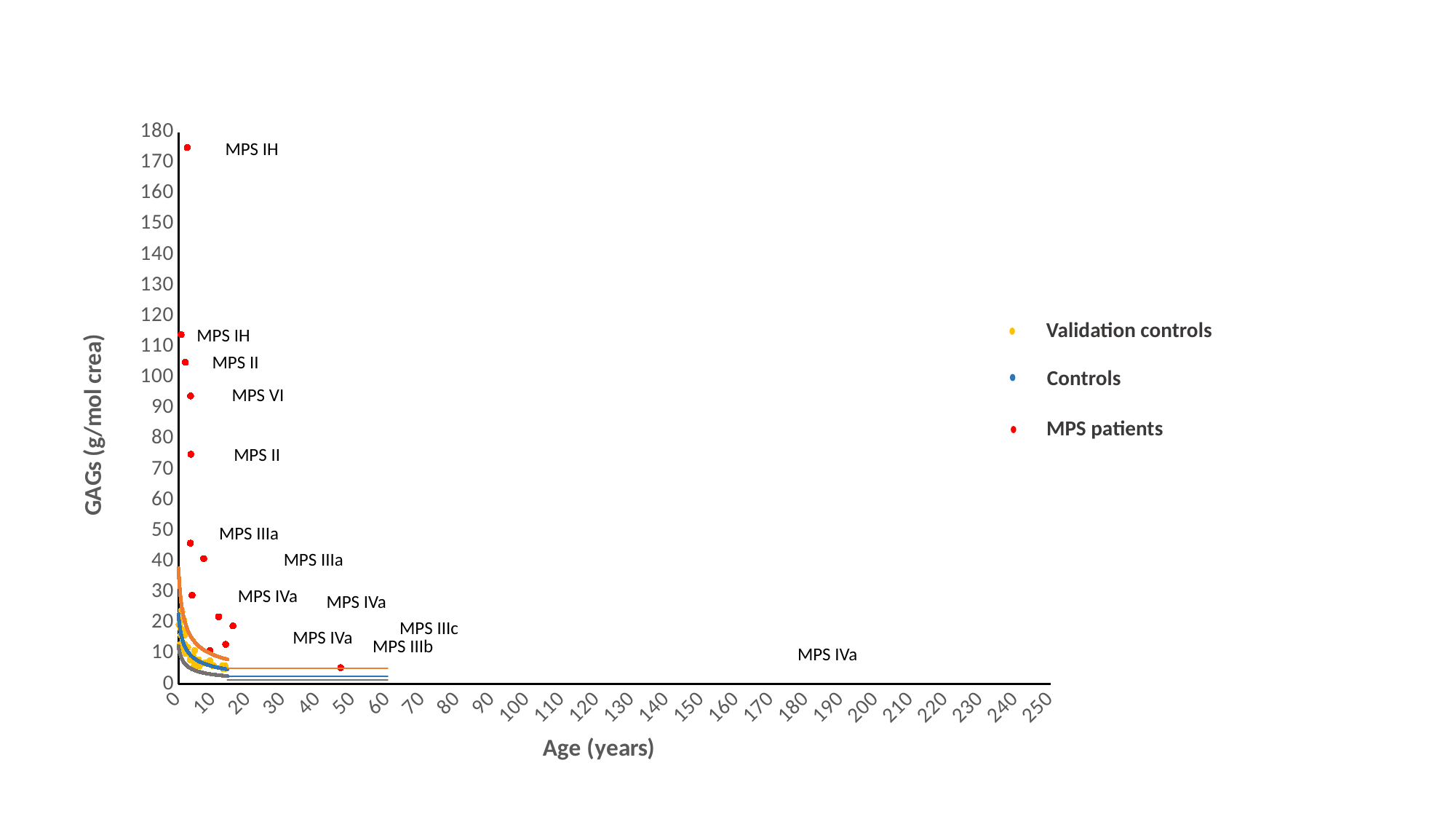

### Chart
| Category | Controls | Reference curve lower limit | Reference curve upper limit | Mean value | | | | | | | | Validation controls (g GAG/mol crea) | |
|---|---|---|---|---|---|---|---|---|---|---|---|---|---|MPS IH
Validation controls
MPS IH
MPS II
Controls
MPS VI
MPS patients
MPS II
MPS IIIa
MPS IVa
MPS IIIc
MPS IVa
MPS IIIb
MPS IVa
