# Supplementary figures and images for: Calculation of continuous reference intervals for biological parameters exhibiting strong age‐dependent level changes: Its application to glycosaminoglycans and sialic acid in urine
Source: JIMD Rep. 2024 Oct 1;65(6):442–9. doi: 10.1002/jmd2.12448 (PMC11540561; doi:10.1002/jmd2.12448)

## Slide 1
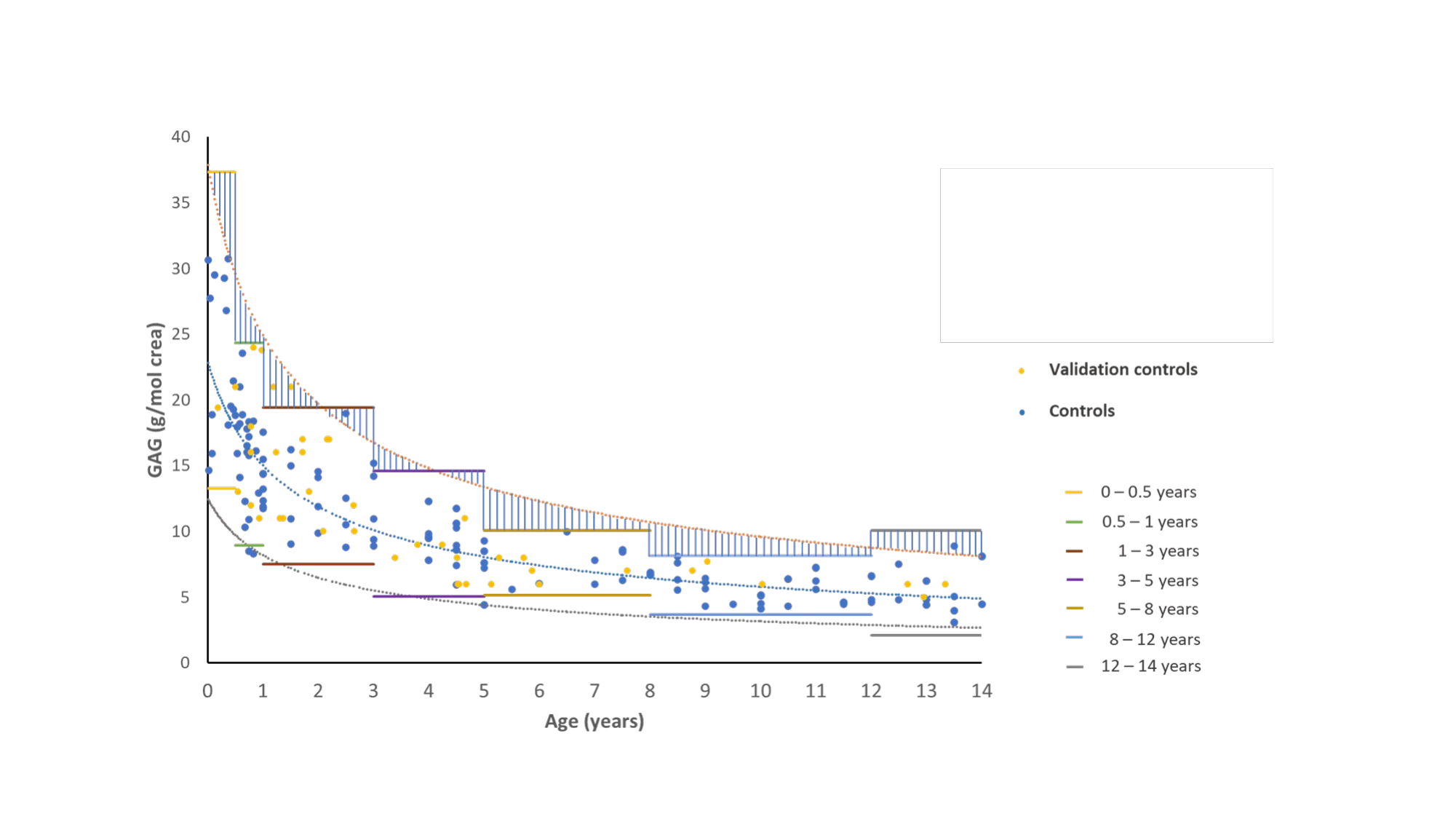

Supplement: Supplementary file 8 — Figure S8. Discrete and continuous reference intervals of GAGs. The striped areas are the areas of discrepancy between the discrete intervals and the continuous interval. [file JMD2-65-442-s006.pptx]
